# In Situ TEM and AFM for Investigation of Materials

**DOI:** 10.3390/ma14092140

**Published:** 2021-04-23

**Authors:** Luciano Lamberti

**Affiliations:** Dipartimento di Meccanica, Matematica e Management, Politecnico di Bari, 70125 Bari, Italy; luciano.lamberti@poliba.it

Materials can be considered the backbone of all technological applications. In fact, the basic question faced by scientists and engineers is how to select the material most suited for the considered scenario. However, material selection must rely on a “complete” (or at least “most detailed as feasible”) knowledge of material properties and expected behavior under the prescribed working conditions. This explains why materials research always gathers the highest interest of scientific community. Material’s behavior in a specific application context often depends also on surface or/and interface properties. For example, surface asperities may strongly affect thermal conduction or electro–chemical behavior as well as tribological properties such as friction and adhesion.

The development of new materials and the use of “traditional” materials in more demanding applications must be supported by efficient investigation techniques. Nowadays, the main concern is to increase measurement resolution to the nanometer or sub-nanometer scale. Transmission electron microscopy (TEM) [1,2] and atomic force microscopy (AFM) [3,4] are two well established techniques for the nanoscale investigation of materials/structures/devices. Assessing nanoscale behavior and understanding how this will affect macroscale behavior may allow exploitation of the full potentiality of the selected material, tailoring it to specific applications or even develop new material concepts starting from currently available technologies.

This Special Issue reports advanced in situ investigations on materials and structures employing TEM and AFM techniques. The issue includes six research articles covering nano-metrology (including damage/defect detection), characterization of surface properties (including tribological analysis), measurement of displacements and strains at the nanoscale (in an SiC crystal with edge dislocations), development of new coating technology for cutting tools, nanoscale mechanical characterization of complex biological structures (e.g., cell membranes).

Cai et al. [5] used atomic force microscopy to investigate nanoscale morphological transformations of silicate glass microchannel plates (MCPs). MCPs are specially designed 2D arrays of microscopic channels for charge particles multiplied, widely used in astronomical, bio-imaging, nuclear science and weak signal detection applications. While hydrogen reduction is the key to determining electrical conductivity of MCPs, no systematic research ever attempted to understand performance variation laws of nanoscale microstructures and surface micro-conductivity in lead silicate glass with respect to reduction temperatures. Effects of hydrogen temperature and nanoscale morphological transformation in modulating physical response of MCPs were quantitatively assessed in [5], thus opening new possibilities to characterize the nanoscale electronic performance of multiphase silicate glass.

An interesting example of how atomic force microscopy can be used for characterizing tribological properties is given in [6]. Hildyard et al. analyzed the nano-tribological behavior of graphene oxide (GO) coatings realized via electrophoretic deposition. They evaluated effect of deposition conditions on frictional parameters of deposited coatings at the nanoscale asperity level. Furthermore, a novel multi-scale numerical approach was developed in [6] to extend coating behavior to macroscale. Shear strength of asperities was determined by using AFM in contact mode. For that purpose, a normal load *N* was applied to the AFM cantilever, measuring the lateral bending of the cantilever as it scans across the coating surface, calculating the frictional force *F* at the load *N*. The slope of the linear branch of the *F*-*N* curve built for different values of *N* was taken as the shear strength of the asperities of the coatings. In summary, Ref. [6] demonstrated how to use AFM for obtaining potentially desirable tribological coatings by assessing the effect of frictional parameters resulting from different deposition conditions.

A very challenging task is to measure displacements and strains at the nanoscale. In this regard, Sciammarella et al. [7] provided experimental evidence supporting the Cauchy–Born conjecture on the relationship between changes in atomic configuration and continuum mechanics solutions. For that purpose, they analyzed high-resolution TEM patterns of a 4HSiC crystalline array containing edge dislocations. The specimen was a thin crystal wafer of about 10 nm thickness under a state of plane-residual-stress generated by the dislocations. The crystalline array was considered as a grating distorted (i.e., modulated) by the dislocations. Fourier analysis was used for determining displacements and their derivatives according to the Eulerian description. These derivatives were given in input to the nonlinear strain tensor of the 4HSiC crystal. Principal strains obtained from the strain tensor diagonalization were finally given in input to the constitutive equations of the crystal to determine stress field near dislocations.

Transmission electron microscopy becomes very useful for damage/defect analysis. For example, contamination (e.g., metal impurities and redeposited compounds) and brittle scratches that may occur in the fabrication of fused silica optics for high-power laser systems should be suppressed or at least mitigated. Noncontact ion beam etching (IBE) is a nanometer/sub-nanometer precision fabrication technology very suited to fused silica optics thanks to the ion sputtering effect. However, research on low-damage fabrication of fused silica optics using IBE is still in the initial stage. In view of this, Zhong et al. [8] used high-resolution TEM to observe subsurface nanoscale damage evolution before and after IBE. It was found that IBE has an evident cleaning effect on optical surfaces.

Pacella et al. [9] investigated the use of ultrasonic consolidation for applying thick coatings (20–100 μm) on ultra-hard polycrystalline cubic boron nitride (PcBN) materials. PcBN are super-hard materials widely used in cutting tools for precision machining of automotive and aerospace parts. In particular, Pacella et al. proposed an innovative coating solution where a multi-walled carbon nanotube (MWCNT) powder is preplaced on a PcBN substrate. Optimum processing parameters (i.e., bonding speed, energy, and pressure) for the MWCNTs to bond to the dissimilar substrate were identified. Sensitivity of mechanical properties of the coated tools was investigated with several techniques including electron microscopy, energy dispersive X-Ray spectroscopy, micro-hardness analyses, and white light interferometry. TEM was specifically used for investigating the structure of the carbon nanotubes (pre coating).

The last article of the Special Issue regarded the mechanical characterization of a complex biological structure, the immature equine zona pellucida (i.e., the extracellular membrane surrounding oocytes) [10]. For that purpose, Ficarella et al. developed a hybrid framework combining AFM nanoindentation experiments, visco-hyperelastic models, nonlinear finite element analysis and nonlinear optimization. The results of the characterization process were fully consistent with experimental evidence. AFM data were fitted by a continuum mechanics model and the identified mechanical properties were interpreted in view of the real material structure detected by electron microscopy. Such a multi-scale approach should be the paradigm of mechanical characterization of highly nonlinear materials such as soft polymers.

The variety of topics covered by this Special Issue should attract readers working in very different fields such as materials science, mechanical/optical/bio-engineering, technology, biology and medicine, chemistry and physics. The miniaturization of technological devices and the growing number of applications entailing the use of nanomaterials and nanostructures certainly go in the direction of an increasingly intensive utilization of atomic force microscopy and transmission electron microscopy techniques.
